# Bibliography

**Published:** 1905-12

**Authors:** 


### Bibliography.

---

The American Text-Book of Operative Dentistry. In contributions by eminent authorities. Edited by Edward C. Kirk, D.D.S., Professor of Clinical Dentistry in the University of Pennsylvania, Philadelphia; Editor of *The Dental Cosmos;* Officier de l'Acadèmie de France. Third edition, revised and enlarged. Illustrated with 875 engravings. Lea Brothers & Co., Philadelphia and New York, 1905.

This, the third edition of the "American Text-Book of Operative Dentistry" has necessitated some changes among the contributors. This seems a wise procedure, in view of the fact that it has become necessary to treat various subjects in dentistry in special publications. While this burdens the library with many books, it relieves, or should relieve to that extent, books supposed to be confined to one special branch. The chapter on Embryology has been omitted and new matter has been added. The very valuable chapter on the "Use of the Matrix in Filling Operations," by Dr. Crenshaw, of Atlanta, will be read with interest and profit. Some of his statements will be antagonized, such as this, quoted from his article: "It will not matter what form of cohesive gold is employed if the heat sufficent to change its molecular arrangement has been applied in annealing the gold. It is practically impossible to adapt it, unaccompanied by linings, so as to secure moisture-proof joints, and therefore permanent results." This seems to the writer an assertion directly contrary to experience. Cohesive gold foil can be laid up against walls so perfectly as to defy the inroads of moisture. To think otherwise would contradict the experience of the past forty-seven years. It all depends upon the manipulator. It is true there is more danger of fillings leaking under this material than with non-cohesive gold, the latter admitting of more careless handling, but that cohesive gold, properly annealed and properly manipulated, will preserve teeth, needs no argument at this period of practice. The author of this chapter is evidently a strong advocate for the use of non-cohesive gold, and the writer is not disposed to criticise this as he fully appreciates its value, having had long experience in both methods of practice; but he must deprecate the condemnation of a property in gold that has met all conditions in tooth structure and saved teeth impossible under older methods.

The chapter on Orthodontia has been changed by substituting Dr. E. H. Angle in the place of the late Dr. Clark L. Goddard. This was arranged previously to the death of the latter and with his "cordial consent."

Dr. Angle's work is so well known and regarded by many, including the editor of this book, as the "chief exponent of the modern trend of thought upon orthodontia as a problem of occlusion," that criticism would be out of place. It is, however, not possible to agree with all his conclusions, and the reviewer must regard some of his ideas as peculiar to his own teaching and not altogether those of some other equally prominent workers in the same direction. Readers of works on orthodontia and some other subjects are getting weary with the constant repetition of illustrations. We have become so familiar with some of Dr. Angle's patients, through the half-tones in this chapter and previous publications, that introductions would be superfluous if circumstances called for meeting them in the social circle. The introduction of the comparatively easily prepared half-tone should have caused the old method of repeating illustrations to become obsolete. It seems, however, to be growing again under a false economy to the very great detriment of interest in authors' productions.

This chapter, however, must not be judged by this criticism. It is but a small part of the 171 pages taken up by the author of the chapter—a book in itself. It is a valuable production and will appeal to the practical man, whether he agrees with all the ideas enunciated, or prefers to permit them still to remain within the domain of theory.

The book, as a whole, must appeal to every practitioner. It is the thought and practice of a number of men, and has a distinct value because of this variety. It loses, necessarily, something of the harmony of statement belonging to a work prepared by a single mind, but gains in a wider observation of the details of practice. In this respect it becomes in a measure the complete history of operative procedures from the earliest periods, and when the comparison is drawn between this book and those published a half century in the past, the progress is marked. In making this statement it must not be understood that the writer regards earlier workers deficient, for this would not be true, but in the systematic arrangement of study, the present period shows a marked advance over the period named, and this is why this volume is an epitome of all the work of the past years.

The publishers have done their part in the preparation of the book with their usual ability. It is a very satisfactory production in paper, type and illustrations where their responsibility rests with the latter.

A Text-Book of Physiology, Normal and Pathological. For Students and Practitioners of Medicine. By Winfield S. Hall, Ph.D., M.D. (Leipzig), Professor of Physiology, Northwestern University Medical School, Chicago; Member of the American Physiological Society; Member of American Association for the Advancement of Science, etc., etc. New (2d) edition, revised and enlarged. In one octavo volume of 795 pages, with 339 engravings and three full-page colored plates. Lea Brothers & Co., Publishers, Philadelphia and New York, 1905.

This, the second edition of this very valuable work of Dr. Hall, carries with it its own recommendation in its carefully prepared pages. The author writes in the preface to the first edition, that "The plan of the work adapts it to the needs of several classes of readers. Medical students will, it is hoped, find a clearly defined exposition of Physiology proper, its relevant facts from Chemistry, Physics, and Morphology and accompanying outlines enabling them to arrange their knowledge in an orderly and logical manner. Students in literary or scientific institutions who are preparing for the study of medicine or of physiology as a specialty, will find the method of the book equally adapted to their needs."

In the preface to this second edition the author has added subchapters on Pathologic Physiology. The reason for this addition is stated to be that "It is becoming apparent to medical educators that to master normal physiology alone without applying its laws to the symptomatology of disease is to miss a large part of the service which physiology should render." That this is true must be clearly evident to every student of the subject.

A careful reading of the book leaves the impression that the author has prepared it from original observations, and although he has properly made use of the investigations of others, his clear explanation of various phenomena indicates the scholarly investigator. This valuable and absolutely necessary trait in an author's character and work at times leads to the fault, by no means uncommon with some teachers, of talking beyond the comprehension of the stupid man in the class. For this reason some parts of this book seem to be better adapted to the practitioner than to the mental capacity of the average student. For the former class it must prove a storehouse of valuable knowledge upon the subject which it treats.

Individuals will take up the study of this book and find interest in those chapters more directly related to their own special studies, and it, therefore, becomes somewhat difficult to point out to our readers any part of it as superior to any other part. The reviewer, while an interested reader of all the chapters, found special satisfaction in reading the one on digestion, covering, as it does, the entire subject from Physiology of Digestion to the Pathologic Physiology of Digestion.

Chapter XI, upon "The Physiology of the Nervous System," is of peculiar value to practitioners of the healing art, as they are brought constantly in contact with the phenomena accompanying nerve lesions. This is peculiarly applicable to dental practitioners. While the latter are not supposed to be deficient in a general knowledge of the nervous system, they can learn much from this scholarly specialist.

In the former chapter on Digestion, the author antagonizes the generally accepted dictum that water should not be taken with meals and his reasons for this are worth quoting. Under the subtitle, "The Influence of Dilution," he says: "Much has been said and written against the drinking of cold water with meals and especially at the beginning of meals. There is more misconception regarding water than any other food. Without entering into a discussion of the details of the question, and without citing the numerous and reliable authorities, the author will briefly state a few of the fundamental facts regarding the relation of water to nutrition: (1) Water is a prime necessity to the animal body. Lack of sufficient water is just as certain to lead to a derangement of the nutrition of the body as is lack of sufficient solid food. Most people use too little water ; few people use too much water. (2) The free use of water does not tend directly to the accumulation of fat. The statement that it does so is a fallacy, which arises from these facts: The free use of water facilitates the processes of nutrition and economizes food by utilizing a greater proportion of it. * * * (3) Cold water stimulates the free secretion of the gastric juice. Cold water in moderate quantity at the beginning of a meal thus hastens the gastric digestion and makes more efficient the antiseptic action of the gastric juice."

Space will not permit quotations from other portions of the book, although the temptation is to give some of the author's conclusions to the reader.

This work of Dr. Hall should appeal to every critical student, for it embraces not the results of patient investigation only, but the reasoning of a philosophical thinker, and the two qualities combined have given a result valuable to the student of the subject, whether he be classed among the undergraduates or among those having had years of experience.

The work is carefully illustrated with engravings and colored plates and the general make-up is creditable alike to author and publisher.

Manual of Chemistry. A Guide to Lectures and Laboratory Work for Beginners in Chemistry. A text-book specially adapted for students in Medicine, Pharmacy and Dentistry. By W. Simon, Ph.D., M.D., Professor of Chemistry in the College of Physicians and Surgeons of Baltimore and in the Baltimore College of Dental Surgery; Emeritus Professor in the Maryland College of Pharmacy, Department University of Maryland. Eighth edition, thoroughly revised. Lea Brothers & Co., Philadelphia and New York, 1905.

This very valuable book is now presented in the eighth edition, a sufficient recommendation in itself without further notice.

The author says in his preface that "This Manual has been delayed for a year in order to incorporate in it the changes and additions of the new Pharmacopœia. * * * Several of the chapters on organic chemistry have been practically rewritten and the matter has been rearranged so to conform to modern views. * * * The request of teachers in dental schools to consider more fully dental metallurgy has been complied with."

This chapter, the fourth in the book, will be found of value to the dental teacher equally with the student, covering, as it does,

"metals and their combinations." It is probable that this will not take the place of special works on metallurgy, of which there are at least two in this country, but it occupies a fitting place in a text-book of chemistry written, in part, for dental students and dental teachers.

The book has been so extensively reviewed in its various editions and with a deserved reputation established, that nothing specially new relating to its value can be written. The author has not deemed it necessary, beyond changes noted, to do much more than endeavor to bring the manual, in all its parts, "up to date."

Every dentist, whether familiar with the subject or not, should have a work of this kind in his library, if not for study at least for reference, and the writer knows of no work better adapted for the latter purpose than the one under consideration.

It contains besides sixty-six illustrations, one colored spectra plate, and there are eight colored plates representing sixty-four chemical reactions.

LONG'S DENTAL MATERIA MEDICA AND THERAPEUTICS. A Text-book of Dental Materia Medica, Therapeutics and Prescription Writing. For Students and Practitioners of Dentistry. By Eli H. Long, M.D., Professor of Materia Medica and Therapeutics in the Dental and Medical Departments of the University of Buffalo, New York. New (2d) edition, enlarged and revised to accord with the new United States Pharmacopœia. In one octavo volume of 298 pages with 7 engravings and 18 colored diagrams. Cloth, $3.00 net. Lea Brothers & Co., Publishers, Philadelphia and New York, 1905.

This, the second edition of Long's Dental Materia Medica and Therapeutics, indicates its value, for a new book on this somewhat overwritten subject must present certain attractive features to make it worth while for students and teachers alike to seek its pages for instruction.

The book was reviewed very fully in the first edition and the writer has nothing more to add to the opinions then expressed, beyond the fact that, upon the whole, he considered it the best text-book on Materia Medica for students in dentistry.

The writer did not agree with the author's method of arranging

"remedies into groups, based upon their action and uses," and he still continues to entertain this opinion. It means annoying reference to index, and that continually, in the effort to find out all the author considers important in the matter of remedies. It is very doubtful whether the "student unconsciously acquires a *comparative* knowledge of drugs and their values" by this arrangement, and it certainly adds to his labor.

While not necessarily agreeing with all the author's views in his therapeutics of the various drugs, it is with exceptional pleasure that the writer has again gone over this portion of the book. It is certainly not the least valuable portion of the 298 pages. It is anticipated that the book must be eventually adopted as a text-book in all dental colleges, for the reason it satisfies a need not heretofore met in the general text-books.

A Practical Treatise on Artificial Crown, Bridge and Porcelain Work. By George Evans, formerly Lecturer on Crown and Bridge Work in the Baltimore College of Dental Surgery, etc. Seventh edition. Revised and enlarged. With 734 illustrations. S. S. White Dental Mfg. Co., Philadelphia, 1905.

The author says of this, the seventh edition of "Artificial Crown, Bridge and Porcelain Work:" The endeavor has been, as in each of those which preceded it, to better meet the requirements of a practical treatise for college and post-graduate study and as well of a reference book for the practitioner. To effect this in the present state of the art has involved a revision so extensive as to make the present edition almost a new work."

That this book now covers 448 pages is sufficient to indicate the wonderful growth of this branch of dental work. When it is considered that the crown of a few years in the past was of simple construction and that bridge work was entirely unknown, it becomes difficult to realize that this branch has now reached practically the proportions of a specialty in dentistry, but such it is. The author of this work has occupied a prominent part in the upbuilding of this branch and is justly regarded as an authority.

The book has been reviewed repeatedly as each edition has appeared and the dental profession is sufficiently familiar with it to accept the fact that it has been thoroughly revised to make a demand for it as a book of reference in every office in this or other countries.

From the reader's point of view it must prove very satisfactory in type and paper, as well as in the care extended by the publisher to make it as perfect as possible in other respects.

THE PHYSICIAN'S VISITING LIST FOR 1906. Fifty-fifth year of its publication. P. Blakiston's Son & Co., Philadelphia.

This well-known visiting list needs no special notice after more than a half century of a satisfactory place in medical practice. While not quite as satisfactory for dental engagements, it has a value in other respects, and could be used for that purpose. Its tables are valuable. The "Dose Table" is worth more than the cost of the visiting list, giving the quantity in apothecaries' weight and metric system. This, if not equally important to the dentist, should be in his hands for ready reference.

